# Effects of Paternal Exposure to Alcohol on Offspring Development

**Published:** 1994

**Authors:** Theodore J. Cicero

**Affiliations:** Theodore J. Cicero, Ph.D., is professor of neuropharmacology in the Department of Psychiatry, Washington University School of Medicine, St. Louis, Missouri

## Abstract

Paternal alcohol consumption may affect fetal development through a direct effect on the father’s sperm or gonads. This possibility casts new light on the heritability of alcoholism in humans.

The adverse consequences of maternal alcohol intake during pregnancy on fetal outcome are well documented (for a review, see Meyer and Riley 1986). However, the possibility that paternal alcohol consumption also may induce deficits in the progeny has received relatively little attention. This is somewhat surprising, as alcoholism appears to be linked genetically with the father in humans (Merikangas 1990; Pickens et al. 1991), and studies indicate that male offspring of alcoholic fathers have behavioral problems and impaired intellectual skills as well as hormonal and nervous system anomalies (see below).

This article discusses the possible direct effects of paternal alcohol consumption on fetal development, distinguishing these effects from studies of the genetic heritability of alcoholism. The article also discusses the possibility that such paternal effects may contribute to cognitive and biochemical disturbances that may be associated with altered responses to alcohol that might lead to addiction.

For purposes of this review, alcoholism is broadly defined as the excessive and repetitive consumption of alcohol that results in significant disturbances in a person’s life, such as preoccupation with drinking to the exclusion of other activities, inability to perform adequately at work, and deterioration of family or other social interactions. In general, the study populations discussed below meet not only these criteria but also others required for a clinical diagnosis of alcoholism.

## Deficits in the Offspring of Male Alcoholics

Many studies have indicated that children of alcoholic fathers often demonstrate impaired cognitive^1^ skills and are more likely to be hyperactive than are children of nonalcoholic biological parents (Hegedus et al. 1984; Tartar et al. 1989). These studies generally adopted controls to ensure that the effects were not due to such factors as maternal drug use, socioeconomic variables, race, and psychiatric or medical disorders in the parents. These effects also were observed in children borne of alcoholic biological fathers but raised by non-alcoholic adoptive parents.

Sons of alcoholics also have abnormal electrical activity in the brain as measured by the electroencephalograph (EEG) (Begleiter and Projesz 1988; Ehlers et al. 1989; Schuckit et al. 1987*a*). Moreover, it has been shown that the sons of alcoholics, when compared with sons of nonalcoholic parents, demonstrate abnormal hormonal responses to short-term administration of alcohol (Schuckit 1988; Schuckit et al. 1987*a*,*b*, 1988). Hence, these data seem to suggest that genetic factors of the biological fathers that relate to their drinking behavior may have a significant effect on the intellectual and behavioral development of their offspring.

## Genetic Basis for Transmission of Alcoholism

The foregoing studies generally represent attempts to identify markers for the predisposition for alcoholism. A marker can most easily be understood as a specific trait that may predict whether a person is at risk for developing a medical disorder. For example, blood tests can be used to predict the occurrence of various genetic disorders, including cystic fibrosis and Down syndrome. Researchers are attempting to identify markers that could serve as early indicators of potential susceptibility to alcoholism.

Genetic linkage studies are a more useful approach for identifying a genetic basis for alcoholism. These sophisticated molecular biological techniques attempt to establish causal links between disorders and specific genes. Using these techniques, researchers have identified genes responsible for at least some types of Alzheimer’s disease, cystic fibrosis, and other genetically transmissible disorders. The discovery of an association between a medical disorder and a specific gene provides a better understanding of the mechanisms underlying the disorder and may therefore provide a basis for improved treatment.

In the case of alcoholism, genetic linkage studies have produced equivocal results. This is not surprising, as the heterogeneous nature of alcoholism is not likely to be explained by a single gene. Furthermore, the results of linkage studies depend heavily on the specific criteria used to define alcoholism in the subject population and the control populations with which they are compared. Most such studies have used widely varying sets of criteria, making comparisons between studies difficult.

### Twin and Adoptee Studies

Results of twin and adoptee studies are consistent with a genetic predisposition to the development of alcoholism. These studies have demonstrated that sons borne of alcoholic biological fathers and raised by nonalcoholic fathers are at much greater risk for developing alcoholism than are sons of nonalcoholics raised by either alcoholic or nonalcoholic fathers (for reviews, see Cloninger et al. 1989; Merikangas 1990; Pickens et al. 1991). Perhaps most importantly, these studies demonstrate that the drinking history of the stepfather is irrelevant in terms of the development of alcoholism in sons borne of either alcoholic or nonalcoholic biological fathers. Sons of nonalcoholic biological fathers had the same incidence of alcoholism as in the general population, whereas sons of alcoholic biological fathers seemed to have a higher incidence of alcoholism irrespective of how they were raised.

Twin and adoptee studies are useful for establishing a familial or genetic basis for alcoholism, but they are limited in scope. Thus, it has not been possible to distinguish completely between environmental and biological factors in the development of alcoholism. Nevertheless, the studies discussed in this and previous sections suggest that there is a high incidence of alcoholism in the offspring of alcoholic fathers and that these offspring can be clearly distinguished from children of nonalcoholics in several ways.

### Alcoholic Mothers and Daughters

As a result of the studies discussed above, it has been widely assumed that alcoholism is genetically transmissible only in males. These results may simply reflect the relatively low incidence of alcoholism in females compared with males (e.g., Cloninger et al. 1989). However, the diagnosis of alcoholism is being made in increasing numbers of women (Johnston et al. 1987), contradicting the earlier belief that alcoholism affects only males.^2^ Further studies are needed to more firmly establish whether the daughters of alcoholic fathers have any higher risk for developing alcohol-related problems than daughters born of nonalcoholic parents.

In addition, not all studies have adequately considered the role of maternal alcoholism or abuse of other drugs; given the established linkage between prenatal alcohol consumption and developmental anomalies such as FAS, for example, these factors must be rigorously controlled for in such experiments. Another question that has been largely ignored is whether the mother’s drinking behavior can influence alcohol consumption patterns in their offspring independently of the drinking habits of the biological fathers or stepfathers.

## Studies in Animal Models

As discussed above, numerous studies clearly suggest impairments in the sons of alcoholic fathers. However, two important questions cannot be conclusively addressed in studies with humans: Are the observed deficits due to biological or to social determinants? Do these deficits represent the toxic effects of alcohol per se or the genetic transmission of specific traits? The use of an animal model permits a direct assessment of whether the paternal consumption of alcohol is a potential toxicant to the developing fetus. Although no animal model could duplicate the complex psychosocial factors that contribute to alcoholism in humans, such models can be extremely helpful in elucidating the biological aspects of alcoholism.

### Teratogenic Effects of Alcohol

The initial reports of infant malformations and mortality resulting from paternal alcohol consumption in animals appeared more than 70 years ago (Stockard and Papanicolaou 1916, 1918*a*,*b*). These studies demonstrated profound alcohol-induced reductions in fertility, gross developmental abnormalities, and considerable levels of infant mortality. These results were initially dismissed, largely on the grounds that if these massive deficits in animals had clinical significance, they would already have been observed in humans. Another view held that as a clear-cut mechanism for these effects could not be demonstrated, the effects themselves must not exist. Moreover, several attempts to replicate these results were unsuccessful (MacDowell et al. 1926; Durham and Woods 1932).

Renewed interest in the effects of paternal drug administration emerged approximately 15 years ago as a result of studies in animal models showing that alcohol influences male sexual performance and fertility, the viability of offspring, and maturation of the fetus and newborn (for a review, see Abel 1992). These effects appeared to be qualitatively and quantitatively different from those observed in FAS. However, a few reports suggested that FAS could occur in offspring of alcoholic fathers with no evidence of heavy alcohol consumption during pregnancy by the mother (Scheiner et al. 1979; Henderson et al. 1981; Randall and Noble 1980). It remains to be resolved whether the anomalies observed in the offspring of fathers exposed to alcohol are characteristic of FAS or some other syndrome. Nevertheless, it seems clear that paternal, pregestational alcohol administration can produce adverse effects in the offspring, at least under the conditions of these early studies.

Unfortunately, methodological problems in this research have made comparisons between studies difficult and definitive conclusions nearly impossible. These problems include very limited numbers of experimental subjects; inappropriate modes of alcohol administration, causing problems with nutrition and stress; variation in the length of alcohol exposure among studies; and whether or not a drug-free interval was provided prior to mating. Moreover, some recent studies, using appropriate alcohol administration regimens and adequate control groups, failed to observe any gross anomalies characteristic of FAS such as were observed in some earlier studies.

### Specific Deficits in the Offspring of Alcoholic Fathers

More recently, we and others have examined the influence of paternal alcohol consumption on offspring under well-controlled conditions in animal models (Cicero et al. 1991*b*; Wozniak et al. 1991; Abel 1989, 1992; Abel and Lee 1988; Abel and Moore 1987; Abel and Tan 1988; Berk et al. 1989).

#### Deficits in Puberty and Sexual Maturation

Initially, we focused on the effects of alcohol on puberty and sexual maturation, because alcoholism and alcohol use are increasing among adolescents (Johnston et al. 1987). We found that alcohol administered to prepubescent male rats significantly affected many primary indicators of puberty and sexual maturation as those rats developed. The alcohol diet was terminated when the animals were sexually mature; all reproductive hormonal indicators then quickly recovered, becoming indistinguishable from non-alcohol-fed control animals 2 to 3 weeks after termination of alcohol exposure. In contrast to these results obtained in immature rats, the effects of alcohol on reproductive hormones in the fully mature animal were transitory and of considerably lesser magnitude.

To confirm that the effects of pre-adolescent exposure to alcohol on reproductive hormones were fully reversible, we mated alcohol-exposed adolescent male rats, in which the effects of alcohol on reproductive hormones had apparently completely dissipated (2 to 3 weeks after termination of alcohol exposure), with drug-naive females. We examined sexual behavior, capacity to mate, and the ability to conceive healthy litters in alcohol-exposed male rats compared with controls. We also examined relatively crude indices of the development of their offspring (“alcohol-sired” rats).

Although pregnancy rates were essentially equivalent when alcohol-exposed and control animals were mated with drug-naive females, the size of the litters was modestly but significantly smaller with the alcohol-exposed males. However, in other respects, such as birth weights, ratio of males to females, mortality rates, and gross developmental features, alcohol-sired offspring were identical with controls. Taken together, the results of the above experiments show that early exposure to alcohol adversely affects puberty and sexual maturation, with essentially complete recovery of reproductive function occurring within 2 to 3 weeks after alcohol withdrawal.

#### Offspring Effects

We more fully characterized the development of the offspring of alcohol-exposed and control animals to determine whether more subtle differences might exist. We found significant disturbances in hormonal function in adult alcohol-sired rats compared with offspring sired by normal male rats. For example, male alcohol-sired offspring had significantly lower levels of testosterone and beta-endorphin as well as lighter seminal vesicles.^3^ We were unable to demonstrate any impairment in reproductive hormone function in female alcohol-sired offspring. However, we found that female—but not male—alcohol-sired offspring had abnormal baseline levels of certain stress-related hormones and responded differently to stress than did control female offspring.

The alcohol-sired offspring in these tests did not differ from controls in terms of body weights measured at various times during development, the appearance of various developmental landmarks, or performance on several developmental tests. However, the adult alcohol-sired males performed poorly on several spatial learning tests; other forms of learning appeared to be relatively unaffected. Female alcohol-sired offspring displayed no significant learning impairments on any test we employed.

Thus, our results indicate pronounced gender-specific hormonal function and behavioral defects in the offspring of fathers exposed to alcohol as adolescents. Perhaps of equal importance, the deficits we observed in alcohol-sired offspring appeared to be selective. We observed no differences in several hormonal systems other than those associated with reproductive hormones and stress nor were there differences on a variety of behavioral tests other than those relying on spatial learning. This selectivity might account for previous reports in which no gross developmental anomalies were observed to result from paternal alcohol exposure.

In addition, these results are highly consistent with the observations in humans, in that offspring of alcoholic fathers, as opposed to offspring suffering from FAS, are not grossly malformed or impaired but have pronounced selective intellectual and functional deficits. Thus, our animal model may prove to be useful for examining deficits derived from paternal alcohol exposure in offspring of human alcoholics.

We are unaware of any reports examining the effects of paternal drug administration using an experimental design similar to the one we used in our studies. However, several researchers have reported that exposure of fully mature male rats to environmental toxicants and drugs, including alcohol, can lead to numerous behavioral, biochemical, and hormonal disturbances in their offspring (for reviews, see Cohen 1986; Joffe and Soyka 1982; Narod et al. 1988). For example, Abel (1989, 1992) found that alcohol administration to male rodents adversely affects the hormonal and cognitive status of the offspring.

Although many studies have reported deficits in alcohol-sired offspring, earlier researchers studied only adult animals and used extremely high doses of toxicants or drugs. Moreover, alcohol exposure continued through conception, and the appearance or functional activity of the sperm was often grossly altered. Many of these studies do not permit a distinction between the chronic effects of alcohol and its acute toxicity with respect to reproductive hormonal function.

In our ongoing studies, we have shown that a period of moderate exposure of the father to alcohol during sexual maturation, followed by a drug-free period sufficient to restore normal hormonal status, resulted in the abnormal development of both male and female offspring. Thus, our results presumably do not reflect the acute effects of alcohol or the consequences of withdrawal but rather some residual effect of early exposure to alcohol during development of the future father. Consequently, the experimental design used in our studies may be useful for examining the possible consequences of heavy alcohol use by human male adolescents on the development of the offspring they bear later as adults.

### Mechanisms of Paternal-Alcohol Effects on Offspring

The mechanisms underlying the deficits observed in alcohol-sired animal offspring are not easily explained. It is, however, clear that the results observed in our studies are due exclusively to paternal alcohol exposure, because they cannot reasonably be ascribed to the female. Specifically, the females were drug naive and matched to control animals in terms of their previous capacity to deliver and nurture healthy litters. Moreover, their offspring developed normally with no evidence of fetal mortality, and post mortem evaluation revealed no clinically significant problems in the females that could account for any of the observed effects.

There are three possible mechanisms for the effect of paternal alcohol consumption on the offspring. First, alcohol may directly affect the characteristics and properties of sperm, perhaps by causing mutations in the sperm’s genetic material. Second, sperm may be “selected” in some way such that only a specific population is functionally intact following prolonged exposure to alcohol. Third, alcohol consumption might alter the chemical composition of semen so as to influence the activity of ejaculated sperm.

In this connection, several recent studies have shown that various drugs, including alcohol, may induce subtle mutations in sperm (Obe et al. 1986; Narod et al. 1988), and long-term alcohol exposure may result in gross abnormalities in the appearance and motility of sperm (for a review, see Abel 1992). In addition, it has been demonstrated that drugs and toxicants accumulate in semen (e.g., Yazigi et al. 1991), and some drugs, such as cocaine, may bind to the sperm surface (Yazigi et al. 1991).

These data suggest that drugs may either impair sperm directly, thereby influencing the development of offspring, or be transported to the ovum via the seminal fluid by physically binding to sperm. Alternatively, alcohol might alter the biochemical and nutritional composition of the seminal fluid, which is necessary for the survival of sperm and to ensure successful conception. If the latter is true, then it can be postulated that the embryo may be exposed to high levels of a toxicant or that seminal substances necessary for facilitating and maintaining the embryo are altered during the earliest stages of development, adversely affecting normal maturation. These possibilities are pure conjecture, as a causal link with birth defects has not been established.

It may be significant that endogenous opioids are synthesized in the testes. Endogenous opioids are morphinelike substances, best known as chemical messengers in the brain. Their functions are not clearly understood, but they generally involve modulation of hormonal systems. Endogenous opioids carry out their functions by binding to specific receptor proteins embedded in the surfaces of cells, thereby ultimately causing chemical changes to occur within those cells. Receptors for endogenous opioids have been found to occur in certain testicular cells and on the surface of sperm.

Therefore, these substances may play a significant role in modulating the production of sex hormones and sperm in the testicles. Although the effects of alcohol on testicular endogenous opioid function are unknown, both short- and long-term alcohol use significantly affect endogenous opioid systems in the brain and other organs.

Alcohol and morphine have similar effects on puberty and sexual maturation (Cicero et al. 1991*a*), and the results of breeding animals exposed to morphine during adolescence with drug-naive females were similar to those observed in animals exposed to alcohol. For example, morphine-sired male offspring had lower serum testosterone and other hormone levels, lower weights of the seminal vesicles, and heavier adrenal glands.^4^ Female morphine-derived adult offspring had significantly higher levels of adrenal stress-related hormones in the blood.

Moreover, Friedler and associates (Friedler 1985; Friedler and Cicero 1988) have demonstrated cognitive deficits in the offspring of morphine-exposed males mated with drug-naive females, similar in some respects to those observed in the offspring of alcohol-exposed males. These similarities between two commonly used drugs, alcohol and opiates, raise several interesting questions, such as whether these drugs act through a common pathway and whether other drugs likely to be used during adolescence would produce similar effects.

## Conclusions

Alcohol consumption by male rats appears to have long-lasting effects on their ability to produce normal progeny. Studies suggest that alcohol itself may be a direct toxicant to sperm, inducing subtle yet marked deficits in the offspring of alcohol-exposed fathers. If true, this will require a reassessment of the numerous studies in humans examining the heritability of specific traits predisposing the offspring to alcoholism. Specifically, it has been assumed that the sons of alcoholics inherit some genetic trait that predisposes them to alcoholism, but few investigators have considered the possibility that these deficits could be due to alcohol’s being a direct gonadal toxicant or teratogenic agent.

Results relative to the paternal effects of alcohol on progeny are still in a very early stage of development. A concerted effort must be made to replicate these findings and to address other important issues, such as: How much alcohol must fathers drink to produce deficits in their offspring? Are the effects observed in the offspring of alcohol-exposed fathers transmitted from one generation to the next? Can these effects be reversed by long-term abstinence of the father prior to conception?

Whereas the paternal effects of alcohol on both male and female offspring appear to be pronounced, no studies as yet suggest that any of the deficits observed in animal models are causally related to the development of alcoholism. Studies are needed to determine whether the observed cognitive and biochemical disturbances are associated with altered responses to alcohol that might lead to addiction. Such studies would also help determine whether animal models are appropriate for examining the causal factors and heritability of alcoholism in humans.

## Footnotes

1 Cognition refers to intellectual functions such as information processing, learning, and memory.

2 Editor’s Note: For more information on the role of genetic factors in the etiology of alcoholism of women, see Kendler, K.S.; Heath, A.C.; Neale, M.C.; Kessler, R.C.; and Eaves, L.J. Population-based twin study of alcoholism in women. *Journal of the American Medical Association* 268(14):1877–1882, 1992.

3 Testosterone is the primary male sex hormone; beta-endorphin is a hormonelike substance in the brain, belonging to the class of endogenous peptides; and the seminal vesicles are sperm-producing tubular structures in the testes.

4 The adrenal glands, located above the kidneys, produce hormones that help regulate various metabolic functions and the response to stress.
